# cGAS–STING pathway in ischemia-reperfusion injury: a potential target to improve transplantation outcomes

**DOI:** 10.3389/fimmu.2023.1231057

**Published:** 2023-09-21

**Authors:** Zijian Chen, Yangqi Liu, Zeying Lin, Weizhe Huang

**Affiliations:** Department of Cardiothoracic Surgery, The Second Affiliated Hospital of Shantou University Medical College, Shantou, China

**Keywords:** allografts, cGAS-STING, ischemia-reperfusion injury, programmed cell death, transplantation, inflammation

## Abstract

Transplantation is an important life-saving therapeutic choice for patients with organ or tissue failure once all other treatment options are exhausted. However, most allografts become damaged over an extended period, and post-transplantation survival is limited. Ischemia reperfusion injury (IRI) tends to be associated with a poor prognosis; resultant severe primary graft dysfunction is the main cause of transplant failure. Targeting the cGAS–STING pathway has recently been shown to be an effective approach for improving transplantation outcomes, when activated or inhibited cGAS–STING pathway, IRI can be alleviated by regulating inflammatory response and programmed cell death. Thus, continuing efforts to develop selective agonists and antagonists may bring great hopes to post-transplant patient. In this mini-review, we reviewed the role of the cGAS–STING pathway in transplantation, and summarized the crosstalk between this pathway and inflammatory response and programmed cell death during IRI, aiming to provide novel insights into the development of therapies to improve patient outcome after transplantation.

## 1 Introduction

The innate immune response mediated by the cyclic guanosine monophosphate-adenosine monophosphate (cGAMP) synthase-stimulator of interferon (IFN) genes (cGAS–STING) pathway has long been the front-line defense against pathogens, such as bacteria, parasites, DNA viruses, or retroviruses (1, 2). However, owing to the sequence-independent identification of double-stranded DNA (dsDNA), relevant research on the cGAS–STING pathway has indicated that cellular function extends beyond resisting the invasion of foreign pathogens, and unnecessary activation by accidental sensing of self-derived DNA or mutations can lead to autoinflammatory diseases (3). For example, STING-associated vasculopathy with onset in infancy (SAVI) commonly develops in patients with gain-of-function mutations in TMEM173 (4, 5). Aicardi-Goutières syndrome mainly presents with an aberrant generation of type I IFN, and accumulation of DNA damage may be an important driver of STING-related inflammatory responses (6, 7).

During organ acquisition, preservation, and transplantation, ischemia-reperfusion injury (IRI) exacerbates damage to donor graft tissues when blood flow is restored after a certain ischemic time. A deficient arterial blood supply invariably leads to a redox imbalance and creates a hypoxic environment in donor graft tissues. Surgical blood reperfusion can lead to severe oxidative damage and an inflammatory response following reoxygenation. This series of events aggravates allograft injury and may lead to primary graft dysfunction, which is associated with high mortality and morbidity (8, 9). The cellular and molecular events that occur during IRI are complex and involve innate immune system activation and programmed cell death (PCD) (10, 11). However, their interplay is still not clearly understood. In the context of limited treatment options, it is urgent to develop less toxic and higher specificity immunosuppressors to better control graft rejection and avoid mortality related to their toxicity (12).

In the present review, we offer an overview of the cGAS–STING pathway and highlight its role in patients who have undergone transplantation. We then summarize the pharmacological basis for targeting the cGAS–STING pathway for treating IRI to explore potential treatment approaches for IRI following transplantation.

## 2 Overview of the cGAS–STING pathway

**Figure 1:**
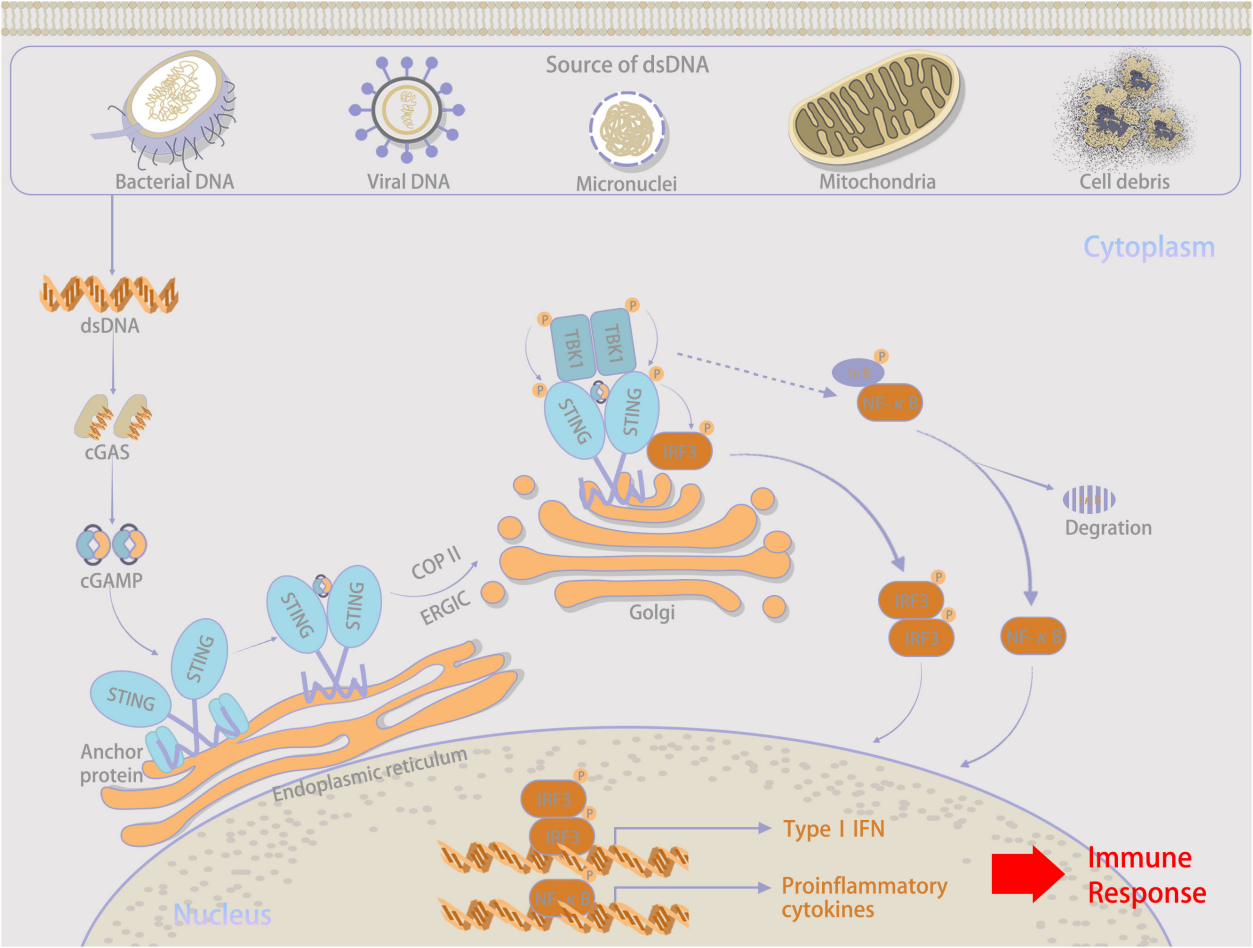
Overall explanations about the cGAS–STIBG signaling pathway.

Cyclic GMP-AMP synthase (cGAS) serves as a novel cytosolic DNA sensor that stimulates IFN production by binding to abnormal DNA within the cytoplasm and activates STING, this activation then triggers host innate immunity in response to “danger signals” (13, 14). The overview of the cGAS–STING signaling pathway is illustrated in **Figure 1**. The binding of abnormally accumulated dsDNA to cGAS in the cytoplasm greatly induces a phase transition, during which cGAS is activated. Recognition is independent of a specific sequence (15–18). Owing to the dsDNA-induced oligomerization of cGAS, a dimerized cGAS–dsDNA complex is catalytically formed (19–21). Activated cGAS promotes conformational changes in the catalytic pocket that allow the cyclization of GTP and ATP as substrates for conversion into cGAMP as a second messenger (22, 23).

**Figure 2:**
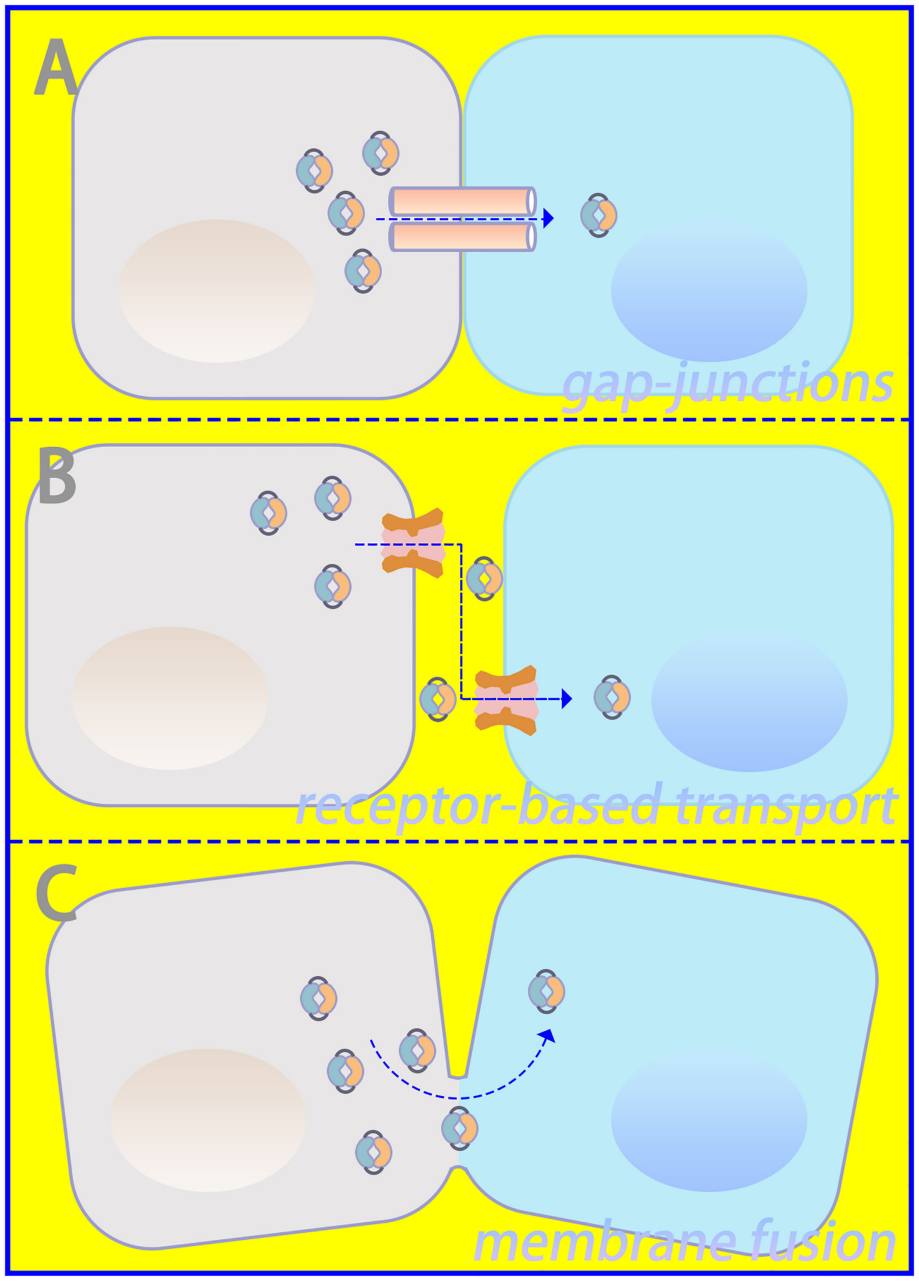
Intercellular communication in cGAS–STING signaling. **(A)** Gap junctions, **(B)** receptor-based transport, and **(C)** membrane fusion could serve as approaches for intercellular transmission of cGAMP.

Important sources of DNA within the cytoplasm include damage-associated DNA released from nuclear and mitochondrial leakage, as well as exogenous pathogen-associated DNA resulting from microbial infection (24, 25). Compared with bacterial cyclic dinucleotides, dsDNA-activated cGAS contains a linear 2’-5’-linked dinucleotide between GMP and AMP that effectively activates human STING (26, 27). Furthermore, higher DNA-binding valences and longer-packed DNA structures facilitate cGAMP production and innate immune signaling (15). Via gap junctions, receptor-based transport, and membrane fusion approaches, activated cGAS can trigger cGAMP transfer from original cells to bystander cells as additional routes to induce downstream signaling cascades (28, 29), which is illustrated in **Figure 2**. Regarding canonical cGAS–STING signaling, cGAMP is produced as an agonist for STING, and its binding to STING located in the endoplasmic reticulum (ER) induces a 180° rotation for ligand binding with the transmembrane domain as a reference, which unlocks the right-handed cross–over connections.

Therefore, during the rearrangement of the STING dimer, the ligand-binding pocket is closed. This important conformational transition enables the oligomerization and release of STING from anchoring proteins, which are further translocated by integrating with cytoplasmic coat protein II complex vesicles (30–32). Cytoplasmic coat protein I-mediated retrograde membrane trafficking is also significant for STING activation (33, 34). Under the assistance of ADP-ribosylation factor GTPases and cytoplasmic coat protein II, higher-order complex STING is transferred from the ER through the ER-Golgi intermediate compartment to the Golgi apparatus. There, STING and the transcription factor IFN regulatory factor 3 (IRF3) are phosphorylated by recruited TANK-binding kinase 1 (TBK1), and nuclear factor κB is activated simultaneously (35–38). Phosphorylated IRF3 further oligomerizes and migrates into the nucleus with nuclear factor κB, and both synergistically initiate the expression of type I IFN and inflammatory cytokines, contributing to the innate immune response (39, 40).

## 3 cGAS–STING pathway and transplantation outcomes

Few studies have tried to elucidate the role of the cGAS–STING pathway in transplantation models. Some preclinical and clinical studies have demonstrated STING as an effectiveness therapeutic target for graft-versus-host disease following allogeneic hematopoietic stem cell transplantation (41). However, research on solid organ transplantation is still in its infancy, and thus more attention should be paid to this field.

The traditional Chinese herbal medicine ingredient ginsenoside Rb3 could alleviate oxidative stress caused by ischemia-reperfusion damage (42, 43). Li et al. used ginsenoside Rb3 to suppress adhesion molecule expression in endothelial cells (ECs) and improve microcirculation of murine transplanted skin flaps. They confirmed that the protective effect of IRI resulted from the inhibition of STING–IRF3 signaling (44). Besides, Yang et al. demonstrated that tumor necrosis factor-α-induced protein-8 like 2 (TIPE2), a negative immunoregulator for immune homeostasis, showed a positive correlation with apoptosis and TIPE2 expression in the graft, which might activate ferroptosis-mediated transplant rejection. TIPE2^−/−^ mice that had undergone heart transplantation experienced insufficient IFN-γ production through the TBK1 signaling axis and increased expression of glutathione peroxidase 4 compared with wild-type mice. Mechanistically, TIPE2 deficiency may inhibit IFN-γ generation in T cells by suppressing the TBK1 signaling axis, prevent lipid peroxidation, and relieve ferroptotic cell death in an injured allograft (45, 46). Mesenchymal stromal cell therapy combined with low-dose tacrolimus is a feasible and safe therapeutic regimen (47, 48). Surprisingly, Chen et al. revealed that combination treatment using low-dose tacrolimus (FK506) and mesenchymal stem cells is beneficial to graft survival, possibly due to weakened graft inflammation by suppressing IFN-γ production and TBK1/IRF3 phosphorylation (49). The generation of STING-deficient mice through gene deletion is more conducive to improving our understanding of the cGAS–STING pathway and its importance in transplantation, and clinical trials are urgently needed.

SAVI is an autoinflammatory disease arising from gain-of-function mutations in the *STING 1* gene from abnormal encoding, leading to the overproduction of type I IFN (5). Three patients diagnosed with SAVI who underwent solid organ transplantation have been reported. The first patient was a 1-year-old infant with SAVI who underwent liver transplantation and immunosuppressive therapy but developed severe multiple biliary cysts and cholangitis in the transplanted liver at the age of 3. Intensive tacrolimus, hydroxychloroquine, prednisolone, and mycophenolate mofetil were administered; however, the patient experienced fatal gastrointestinal bleeding 1 year later (50). The second patient was a 34-year-old woman with SAVI who underwent double-lung transplantation but experienced acute primary graft dysfunction, with acute liver and systemic vasculature complications; she finally died from multiple organ failure (51). The last patient was a 17-year-old girl with SAVI who underwent lung transplantation and developed systemic inflammatory symptoms within 4 months; she was treated with three immunosuppressors, including mycophenolate mofetil, tacrolimus, and prednisolone; however, her symptoms relapsed during prednisolone dose reduction (52). Despite reporting on individual cases, these studies indicated that the abnormal activation of STING in patients receiving transplantation probably led to extremely poor outcomes, even when immunosuppressive therapy was administered. Although the relationship between SAVI and the cGAS–STING pathway is not yet clear, inhibiting STING may be beneficial for improving the outcome of transplant recipients. However, research into this area is still lacking and is considered an important area for future studies.

## 4 Crosstalk between cGAS–STING pathway and IRI

### 4.1 IRI: an important player in allograft injury

**Figure 3:**
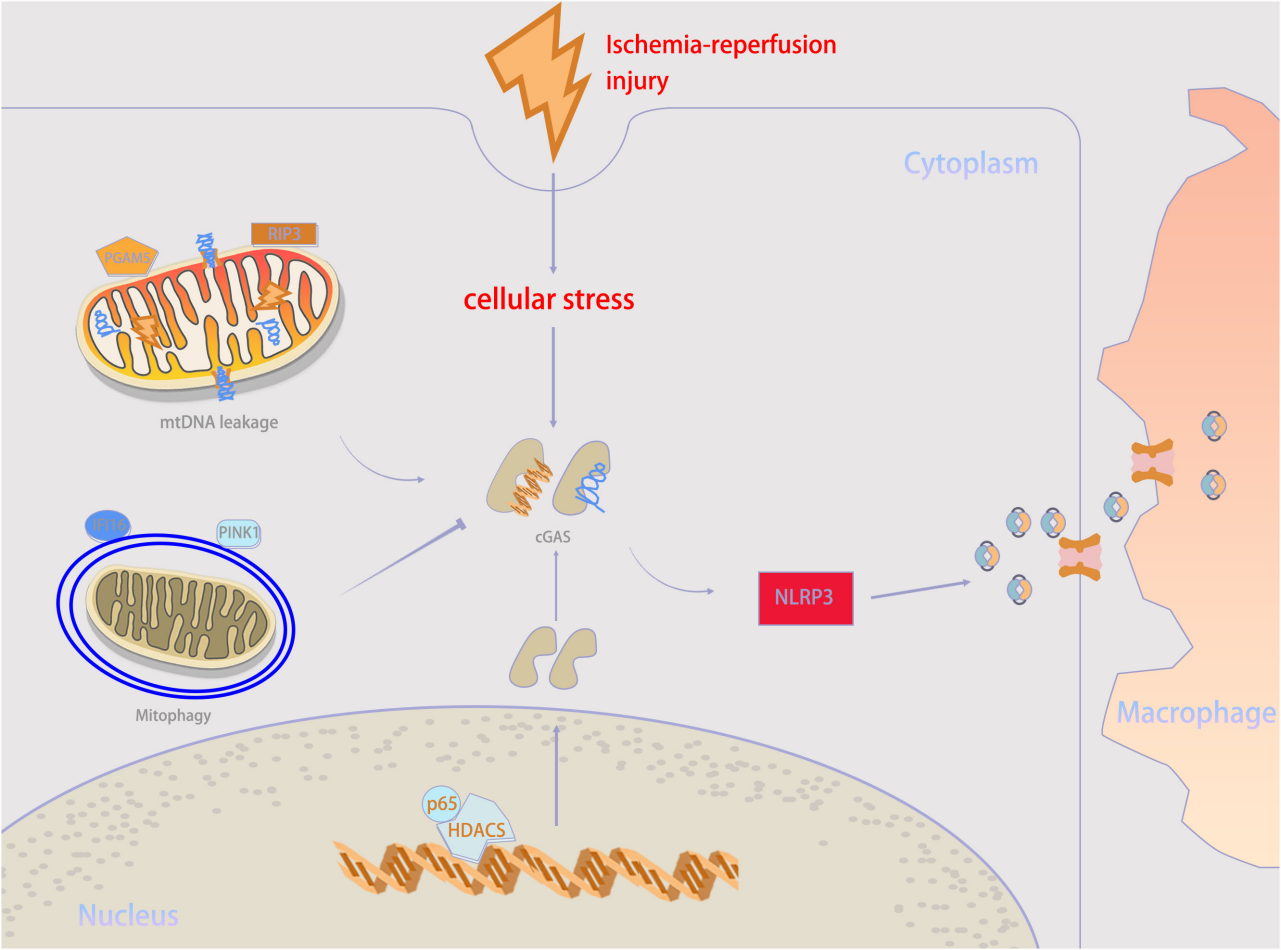
Mechanisms underlying cGAS–STING activity in ischemia–reperfusion injury (IRI) condition. When IRI happens, self-DNA recognition is the primary determinant for cGAS–STING activity. mtDNA leakage, which is executed by RIP3 and PGAM5, is an important source of cGAS stimulation during IRI. But mitophagy mediated by PINK1 and IFI16 help to counteract mtDNA stress. Besides, HDAC3 can upregulate cGAS expression. cGAMP can be transmitted from parenchymal cells to neighboring macrophages through intercellular transmission to activate immune response. PGAM5, phosphoglycerate mutase family member 5; RIP3, receptor-interacting protein 3; IFI16, interferon gamma-inducible protein 16; PINK1, PTEN-induced kinase 1; NLRP3, NOD-like receptor protein 3.

IRI is a major transplant issue, mostly because there is still no effective treatment plan. Recently, it has been demonstrated that targeting the cGAS–STING pathway may be a feasible approach to improve transplantation outcomes by alleviating IRI. In general, the inflammatory response and PCD following ischemia and reperfusion play vital roles in triggering transplant rejection (53, 54). Both transplantation and non-transplantation models of IRI share these key pathogenic mechanisms to increase the incidence rate and mortality, and the cGAS–STING pathway participates in their regulation (2, 55). This suggests a possible relation between post-transplant IRI and the cGAS–STING pathway. Therefore, we have summarized the regulatory mechanisms of the cGAS–STING pathway in IRI in **Figure 3**; **Table 1**, providing new insights for the development of new treatment strategies. Besides, we extracted potential therapeutic agents for treating IRI that may help improve transplant prognosis.

**Table 1:** cGAS-STING pathway-based regulation involved in IRI-related mechanism.

Mechanism	Signaling axis/Trigger	Involved cell/organelle	Regulation description	Impact on IRI	Ref.
Innate immunity-mediated inflammatory response	PGAM5-Mitofilin-cGAS-STING	Mitochondria	Promote DAMP sensing	Proinflammatory	(56)
	RIP3–Mitofilin-cGAS-STING-p65	Mitochondria	Promote DAMP sensing	Proinflammatory	(57)
	PINK1-cGAS-STING	Macrophage	Promote PINK1-induced mitophagy to suppress STING-mediated inflammatory	Anti-inflammatory	(58)
	eCIRP-STING	Macrophage	Promote DAMP sensing	Proinflammatory	(59)
	HDAC3-p65-cGAS-STING	Microglial cell	Promote DAMP sensing	Proinflammatory	(60)
	STING-NLRP3	Macrophage	Upregulate NLRP3	Proinflammatory	(61)
	STING-TBK1/AMPK	Macrophage	Upregulate AMPK Downregulate HIF-1α	Anti-inflammatory	(62)
	mtDNA-STING	Microglial cell	Promote DAMP sensing	Proinflammatory	(63)
	dsDNA-cGAS-STING	T cells	Promote DAMP sensing	Proinflammatory	(64)
	IFI16-STING-NF-κB	Mitochondria	Promote mitophagy, EC activation	Anti-inflammatory	(65)
	cGAS-STING-ERS	Lung epithelial type II cell	Promote ERS	Proinflammatory	(66)
	TXNIP-NRF2-OASL1- STING/TBK1	Macrophage	Upregulate OASL1 to promote STING-mediated TBK1 activation	Proinflammatory	(67)
	STING-AMPK	Unavailable	Upregulate AMPK	Anti-inflammatory	(68)
	STING-mediated lipid peroxidation	BMDM	Promote lipid peroxidation	Proinflammatory	(69)
Programmed cell death	cGAS-STING-Bcl−2/Bax/Caspase−3	Cardiomyocyte	Activate Bcl−2/Bax/Caspase−3	Anti-apoptosis	(70)
	miR-24-3p-STING-IRF3	Hepatocyte	Upregulate miR-24-3p to downregulate STING	Anti-apoptosis	(71)
	cGAS-STING-NCOA4	Neuron	Upregulate NCOA4	Pro-ferritinophagy	(72)
	PI3K-PKB-cGAS-STING	Neuron	Activate PI3K-PKB pathway to suppress cGAS-STING-mediated over autophagy	Pro-autophagic cell death	(73)
	25-HC-mTOR/STING	Neuron	Downregulate mTOR/STING to suppress STING-mediated over autophagy	Anti-autophagic cell death	(74)
	cGAS-mediated regulation of autophagy	Hepatocyte	Regulate autophagy	Anti-autophagic cell death	(75)
	TBK1-FMR1	Renal tubular epithelial cells	Upregulate FMR1	Anti-ferroptosis	(76)
	mtDNA-STING-IFN/TNF-α	Intestinal endothelial cell	Upregulate IFN, TNF-α	Pro-necroptosis	(77)
	STING-calcium-dependent caspase 1-GSDMD	Macrophage	Upregulate calcium-dependent caspase 1-GSDMD	Pro-pyroptosis	(78)

BMDM, bone marrow derived macrophage; STING, stimulator of interferon genes; TBK1, TANK-binding kinase 1; TXNIP, thioredoxin-interacting protein; HIF-1α, hypoxia-inducible factor-1 alpha; AMPK, AMP-activated protein kinase; NLRP3, nucleotide-binding domain and leucine-rich repeat containing protein 3; eCIRP, extracellular cold-inducible RNA-binding protein; DAMP, damage-associated molecular pattern; IFI16, interferon gamma- inducible factor 16; EC, endothelial cell; cGAS, cyclic GMP–AMP synthase; ERS, endoplasmic reticulum stress; 25-HC, 25-Hydroxycholesterol; IRI, ischemia-reperfusion injury.

### 4.2 Inflammatory response in IRI

Although it is yet to be fully explored, it would not be surprising to find that the inflammatory response of post-transplant IRI can be triggered by cGAS–STING pathway. Damage-associated molecular patterns (DAMPs) induced by injury are recognized by cGAS, which trigger immune-mediated inflammation. In general, targeting cGAS–STING pathway may help reduce the inflammatory response in transplantation models.

Increased cytosolic DNA can be recognized by the pathogen recognition receptor cGAS and trigger STING activation-induced inflammation, especially for mitochondrial DNA (mtDNA). Elevated mtDNA accumulation caused by IRI is related to delayed graft function (24, 79, 80). Phosphoglycerate mutase 5-mediated Bax dephosphorylation triggers mtDNA release, activating the cGAS–STING pathway and causing acute kidney injury following IRI (56). Furthermore, kidney IRI increases receptor-interacting protein 3 levels to facilitate mtDNA damage and leakage and then activates the cGAS–STING–p65 pathway by promoting cytosolic mtDNA expression and increases the transcription of pro-inflammatory factors (57). In contrast, mixed lineage kinase domain like (MLKL) pseudokinase knockout significantly enhances PTEN-induced kinase 1-mediated mitophagy activation to alleviate oxidative stress in hepatocytes, thereby inhibiting macrophage cGAS–STING activation and liver IRI (58). These make mtDNA an important target for inhibiting cGAS–STING pathway activation. In addition, a flap endonuclease I inhibitor has been shown to inhibit mtDNA fragment release and cGAS–STING pathway activation (81), which should be further verified in transplantation models. Notably, IRI, especially for apoptosis, is often associated with mitochondrial damage and mtDNA release, shaping a positive feedback circuit (82, 83). Thus, combination therapy for inhibiting cGAS–STING signaling and concurrent mtDNA fragment release may have a synergistic effect. Nowadays, some preclinical studies have made progress. STING inhibitor H-515 can prevent extracellular cold-inducible RNA-binding protein, a potent DAMP, from activating STING and causing intestinal and distant organ injuries (59). Moreover, by transcriptionally upregulating cGAS expression, histone deacetylase 3 (HDAC3) activates the cGAS–STING pathway in a p65-dependent manner; tissue inflammation and injury are triggered; accordingly, HDAC3 inhibitors, such as trichostatin A and MS275, can reverse this detrimental effect (60). Post-translational modifications of cGAS play critical roles in regulating its activity and stability (84, 85), and thus regulation of cGAS expression and function may be another intervention approach.

Recently increasing attention on macrophage-mediated innate immunity as a crucial player in allograft injury (86). Editing macrophage effector function may be an adjuvant therapy to alleviate inflammation. At present, most studies on the regulation of macrophages mediated by the cGAS-STING pathway have set liver IRI as the main research object. Aged mice are more susceptible to aggravated hepatic injury following ischemia-reperfusion, and stronger NLRP3 activation and pro-inflammatory activity of macrophages. Probably because aged parenchyma cells have more extracellular DNA than younger ones, which triggers a stronger STING/TBK1 signaling in macrophages. Consistent with previous studies, older donors are associated with reduced recipient and graft survival rates (61, 87, 88). Knockout of STING in Myelocyte can reduce liver IRI and inflammatory response, indicating that STING activation may promote the proinflammatory response of Monocyte derived macrophages in liver transplantation (62). In addition, Kupffer cells act as tissue-resident macrophages in the liver, playing an important role in in liver IRI as well, but the effect of cGAS-STING pathway toward it is still not clear. However, the promotion of microglial cell M1 polarization can be attenuated by the STING inhibitor C-176 during IRI-induced mtDNA release (63).

It is well known that T cell-mediated immune response is closely related to post-transplant IRI, which is an important factor affecting transplantation prognosis. The cGAS-STING pathway activates adaptive T cell responses by regulating dendritic cells and macrophages (64). By using cGAS-deficient donor tissue, the activation of CD8^+^ T cells in the graft and the proportion of effector memory lymphocytes in the spleen were reduced, and the graft survival was significantly prolonged, this provided a basis for immunosuppressive therapy targeting T cells (65). In addition, the induction of transplantation tolerance depends on the presence of Treg. Surprisingly, activation of the cGAS-STING pathway can induce an increase in the production of regulatory cytokine IL-10 and promote the inhibitory activity of Treg. Therefore, damage to grafts caused by T-cell-mediated adaptive immunity triggered by the cGAS-STING pathway may be the result of immune imbalance (89). Interestingly, although it has been speculated that STING gain-of-function mutations cause disease through abnormal type I interferon signaling, another study suggests that T cell-mediated adaptive immunity may be the main pathogenic factor, but more researches are needed to confirm it (90).

ECs form the primary barrier between the host and solid organ allografts and are essential for inducing cell-mediated acute rejection following transplantation (91). Mitochondrial exposure may upregulate EC adhesion molecules and enhance inflammatory responses by activating ECs (92). Mitochondrial transplant was able to reduce the risk of primary graft dysfunction in lung transplant recipients during ex-vivo lung perfusion (93). Mitochondrial transplantation therapy has shown promise as a therapy in clinical practice, but there is still a lack of research on the underlying molecular mechanisms. A recent study suggested that exposing murine heart ECs to exogenous mitochondria triggers internalized mitochondria-activated IFI16–STING–NF-κB signaling. Subsequently, STING-dependent mitophagy stabilized the endothelium and weakened apoptotic activity, and activated ECs promoted T-cell-mediated co-stimulation blockade-resistant rejection (94); the cGAS–STING pathway possibly plays a significant role in this.

Communication between the ER and mitochondria bridges ER stress and activates the innate immune system (66). Inhibition of the cGAS–STING pathway suppresses ER stress, thereby attenuating lung IRI (95). Surprisingly, ER stress-induced NLRP3 inflammasome activation is possibly a pivotal driver during post-transplant IRI (67). Moreover, the macrophage TXNIP-mediated CYLD-NRF2-OASL1 axis possesses a regulatory effect, and TXNIP disruption suppresses STING-mediated TBK1 activation and subsequent inflammation (96). However, these regulatory effects on macrophages should be further verified in transplantation models.

The cGAS–STING pathway is also involved in controlling energy metabolism. Activated cGAS–STING signaling is accompanied by systemic and cellular metabolism abnormality, involving increased nutrient metabolism and decreased mitochondrial respiration (68). In adipocytes, TBK1 attenuates AMP-activated protein kinase (AMPK) activation to increase energy reserves but inhibits respiration, and promotion of tissue inflammation can be observed in adipocyte-specific TBK1 knockout models (97). Surprisingly, by activating AMPK signaling, the STING inhibitor C-176 improves intestinal IRI-induced acute lung injury (98). Notably, metabolic disorders are often accompanied with intensive mitochondrial damage (69). This indicates the need to determine whether modulation of cGAS–STING pathway-induced metabolism is beneficial to inflammation regulation.

### 4.3 Programmed cell death in IRI

The cGAS–STING pathway participates in a variety of cell death pathways, including pyroptosis, ferroptosis, necroptosis, apoptosis, and autophagy, but without obvious specificity. During IRI following transplantation, multiple types of PCD may coexist. The cGAS–STING pathway serves as a target to provide further insight into their relation.

A strong STING signaling is associated with apoptosis induction (99, 100). Scutellarin plays a protective role in IRI through downregulation of the NLRP3 inflammasome, and it inhibits Bcl−2/Bax/Caspase−3 and the cGAS–STING pathway to ameliorate graft dysfunction and apoptosis (101, 102). STING antagonists can probably be used in combination with it in transplant models. Additionally, the *in vitro* upregulation of miR-24-3p relieves cardiomyocyte apoptosis following IRI (71). This protective effect may be due to the targeting of STING by miR-24-3p to salvage the STING–IRF3 activation-mediated inflammatory response and cellular apoptosis (103). Currently, there is a lack of non-invasive biomarkers in clinical practice that can be used to predict transplant prognosis (72). Although significant miRNAs in tissues are hardly as useful as non-invasive biomarkers, they can be used to inspire later research on biological fluids. More studies are necessary to discover the correlation between miRNAs and transplantation prognosis.

Recent studies have indicated that autophagy is involved in IRI regulation. Direct regulation of autophagy alleviates IRI following transplantation (104). Ferritinophagy is a type of autophagy that targets ferritin to maintain balanced intracellular iron levels. NCOA4-mediated ferritinophagy plays a vital role in IRI, and suppression of the cGAS–STING pathway can diminish ferritinophagy, thus ameliorating IRI (105). Activin A, a well-known neuroprotective factor, is also involved in IRI alleviation through inhibition of cGAS/STING-mediated autophagic cell death (73). 25-hydroxycholesterol is an oxidized cholesterol associated with the pathophysiological pathways of cholesterol homeostasis, immune response, or cell survival, it alleviates IRI by inhibiting STING and excessive autophagy-induced cell death (74, 106). Combinations of these agents with STING antagonists may probably help to lower doses of immunosuppressive drugs and reduce toxicity. Interestingly, cGAS-mediated autophagy has been shown to relieve liver IRI, with this novel protective effect being independent of STING (75). The cGAS–STING pathway participates in the regulation of autophagic cell death in a bidirectional manner. These conflicting results may be related to the existence of multiple noncanonical cascades (76). Thus, future studies should aim to validate the precise mechanism involved in selective activation and understand whether it is equally applicable to other pathogenic process.

Ferroptosis is triggered under conditions of excessive oxidative stress, such as IRI (107, 108). Ferroptotic cell death contributes to inflammatory responses following transplantation (109). Mechanistically, this PCD is induced by activation of the cGAS–STING pathway via lipid peroxidation, this damage can be reversed by the anti-lipid peroxidation drug liproxstatin-1 (110). Surprisingly, lipid peroxidation induced by cellular stress also specifically weakens the STING pathway (70). Thus, more accurate experiments are required to explain their relationship. Additionally, Ubiquitin-specific protease 7 inhibition could reverse ferroptosis-induced IRI, probably because of the suppression of TBK1 degradation and DNA methyltransferase 1 (DNMT1)-mediated methylation of FMRP translational regulator 1 (77, 111).

Both pyroptosis and necroptosis are inflammatory forms of cell death (112, 113). The cGAS–STING–IFN pathway is responsible for maintaining mixed lineage kinase domain-like pseudokinase (MLKL) expression, which is a key component for initiating necroptosis (114). Interestingly, mtDNA released from ECs during intestinal IRI activates the STING pathway and triggers necroptosis through collaborative IFN and tumor necrosis factor-α signaling (115). The cGAS–STING–NLRP3 axis has been demonstrated to be the default mode of inflammatory body activation and pyroptosis. STING activation induces lysosomal cell death and triggers the classic mode of NLRP3 activation (78). STING deficiency in macrophages can inhibit pyroptosis and the subsequent intense inflammatory response during liver IRI; this protective effect is probably due to reduced calcium-dependent caspase 1-GSDMD processing in macrophages (116). However, these results need to be confirmed and complemented in transplantation models.

## 5 Outlook and future perspective

At present, immunosuppressors effectively control transplant rejection; however, considerable issues, such as opportunistic infections, higher occurrence of malignancy, and drug toxicity, have been linked to their use. Compared to immunosuppressor, the breadth of the cGAS-STING pathway in inflammation and PCD is its most powerful advantage, so it has the potential to serve as a multifunctional therapeutic target. Because the mechanism of IRI after transplantation is very complex, single immunosuppressor is limited and combinations are required to achieve the desired therapeutic effect. Superimposed drug toxicity inevitably deteriorates the prognosis. Among them, the most serious side effect of Immunosuppressive drug in transplantation is the severe infection caused by the excessively low immunity of the body (117). The activation of the cGAS-STING pathway has a highly collaborative characteristic, and partial rather than complete blockade seems sufficient to produce anti-inflammatory effects, and proper activity of the cGAS-STING pathway is allowed under obvious infection conditions. Therefore, it is feasible to retain necessary ability of anti-infection while achieve anti-inflammatory, and achieving this balance can help improve prognosis. In order to achieve this balance, a key aspect in the future is to better understand the minimum level of inhibition required for therapeutic benefits. In addition, personalized treatment is the biggest problem faced by the clinical application of immunosuppressors. A lot of efforts are being made to develop small molecule inhibitors targeting the cGAS-STING pathway, and precise treatment based on this pathway may become an important component of future clinical organ transplantation (118).

## 6 Conclusion

IRI is currently a serious complication after transplantation, mainly because there is still no effective therapy to manage it. Attempting to utilize the cGAS–STING pathway as a potential target can provide new insights and help develop treatment approaches for post-transplant IRI. So far, many drugs targeting the cGAS–STING pathway have played therapeutic roles in IRI based on the mechanisms of inflammation and PCD. The next step may include further analysis of the results of these agents in transplantation models and exploring more convincing evidence to elucidate their clinical translation value. In addition, the detailed molecular mechanism of the cGAS–STING pathway is not yet clear, and preventing unexpected and adverse cascade reactions are also issues that need to be addressed.

## Author contributions

Conception and design: ZC and WH. Administrative support: WH. Collection and assembly of data: ZC, YL, and ZL. Data analysis and interpretation: ZC and YL. Drafting the article or revising it critically for important intellectual content: All authors. Final approval of manuscript: All authors. Accountable for all aspects of the work in ensuring that questions related to the accuracy or integrity of any part of the work are appropriately investigated and resolved: All authors.

## References

[1] Cheng Z Dai T He X Zhang Z Xie F Wang S. The interactions between cGAS-STING pathway and pathogens. Signal Transduct Target Ther (2020) 5:91. doi: 10.1038/s41392-020-0198-7 32532954 PMC7293265

[2] Zhang X Bai XC Chen ZJ. Structures and mechanisms in the cGAS-STING innate immunity pathway. Immunity (2020) 53:43–53. doi: 10.1016/j.immuni.2020.05.013 32668227

[3] Harapas CR Idiiatullina E Al-Azab M Hrovat-Schaale K Reygaerts T Steiner A. Organellar homeostasis and innate immune sensing. Nat Rev Immunol (2022) 22:535–49. doi: 10.1038/s41577-022-00682-8 35197578

[4] Jeremiah N Neven B Gentili M Callebaut I Maschalidi S Stolzenberg MC. Inherited STING-activating mutation underlies a familial inflammatory syndrome with lupus-like manifestations. J Clin Invest (2014) 124:5516–20. doi: 10.1172/JCI79100 PMC4348945 25401470

[5] Liu Y Jesus AA Marrero B Yang D Ramsey SE Sanchez GA. Activated STING in a vascular and pulmonary syndrome. N Engl J Med (2014) 371:507–18. doi: 10.1056/NEJMoa1312625 PMC4174543 25029335

[6] Giordano AMS Luciani M Gatto F Abou Alezz M Beghè C Della Volpe L. DNA damage contributes to neurotoxic inflammation in Aicardi-Goutières syndrome astrocytes. J Exp Med (2022) 219:e20211121. doi: 10.1084/jem.20211121 35262626 PMC8916121

[7] Crow YJ. Type I interferonopathies: mendelian type I interferon up-regulation. Curr Opin Immunol (2015) 32:7–12. doi: 10.1016/j.coi.2014.10.005 25463593

[8] Chouchani ET Pell VR James AM Work LM Saeb-Parsy K Frezza C. A unifying mechanism for mitochondrial superoxide production during ischemia-reperfusion injury. Cell Metab (2016) 23:254–63. doi: 10.1016/j.cmet.2015.12.009 26777689

[9] Roesel MJ Sharma NS Schroeter A Matsunaga T Xiao Y Zhou H. Primary graft dysfunction: the role of aging in lung ischemia-reperfusion injury. Front Immunol (2022) 13:891564. doi: 10.3389/fimmu.2022.891564 35686120 PMC9170999

[10] Tang Q Dong C Sun Q. Immune response associated with ischemia and reperfusion injury during organ transplantation. Inflamm Res (2022) 71:1463–76. doi: 10.1007/s00011-022-01651-6 PMC9653341 36282292

[11] Linkermann A Hackl MJ Kunzendorf U Walczak H Krautwald S Jevnikar AM. Necroptosis in immunity and ischemia-reperfusion injury. Am J Transplant (2013) 13:2797–804. doi: 10.1111/ajt.12448 24103029

[12] Parlakpinar H Gunata M. Transplantation and immunosuppression: a review of novel transplant-related immunosuppressant drugs. Immunopharmacol Immunotoxicol (2021) 43:651–65. doi: 10.1080/08923973.2021.1966033 34415233

[13] Sun L Wu J Du F Chen X Chen ZJ. Cyclic GMP-AMP synthase is a cytosolic DNA sensor that activates the type I interferon pathway. Science (2013) 339:786–91. doi: 10.1126/science.1232458 PMC3863629 23258413

[14] Sun Z Hornung V. cGAS-STING signaling. Curr Biol (2022) 32:R730–4. doi: 10.1016/j.cub.2022.05.027 35820380

[15] Du M Chen ZJ. DNA-induced liquid phase condensation of cGAS activates innate immune signaling. Science (2018) 361:704–9. doi: 10.1126/science.aat1022 PMC9417938 29976794

[16] Xie W Lama L Adura C Tomita D Glickman JF Tuschl T. Human cGAS catalytic domain has an additional DNA-binding interface that enhances enzymatic activity and liquid-phase condensation. Proc Natl Acad Sci USA (2019) 116:11946–55. doi: 10.1073/pnas.1905013116 PMC6575157 31142647

[17] Civril F Deimling T de Oliveira Mann CC Ablasser A Moldt M Witte G. Structural mechanism of cytosolic DNA sensing by cGAS. Nature (2013) 498:332–7. doi: 10.1038/nature12305 PMC3768140 23722159

[18] Yang H Wang H Ren J Chen Q Chen ZJ. cGAS is essential for cellular senescence. Proc Natl Acad Sci U.S.A. (2017) 114:E4612–20. doi: 10.1073/pnas.1705499114 PMC5468617 28533362

[19] Li X Shu C Yi G Chaton CT Shelton CL Diao J. Cyclic GMP-AMP synthase is activated by double-stranded DNA-induced oligomerization. Immunity (2013) 39:1019–31. doi: 10.1016/j.immuni.2013.10.019 PMC3886715 24332030

[20] Kranzusch PJ Lee ASY Wilson SC Solovykh MS Vance RE Berger JM. Structure-guided reprogramming of human cGAS dinucleotide linkage specificity. Cell (2014) 158:1011–21. doi: 10.1016/j.cell.2014.07.028 PMC4157622 25131990

[21] Kranzusch PJ Lee AS Berger JM Doudna JA. Structure of human cGAS reveals a conserved family of second-messenger enzymes in innate immunity. Cell Rep (2013) 3:1362–8. doi: 10.1016/j.celrep.2013.05.008 PMC3800681 23707061

[22] Gao P Ascano M Wu Y Barchet W Gaffney BL Zillinger T. Cyclic [G(2’,5’)pA(3’,5’)p] is the metazoan second messenger produced by DNA-activated cyclic GMP-AMP synthase. Cell (2013) 153:1094–107. doi: 10.1016/j.cell.2013.04.046 PMC4382009 23647843

[23] Wu J Sun L Chen X Du F Shi H Chen C. Cyclic GMP-AMP is an endogenous second messenger in innate immune signaling by cytosolic DNA. Science (2013) 339:826–30. doi: 10.1126/science.1229963 PMC3855410 23258412

[24] Ablasser A Chen ZJ. cGAS in action: Expanding roles in immunity and inflammation. Science (2019) 363:eaat8657. doi: 10.1126/science.aat8657 30846571

[25] Landman SL Ressing ME van der Veen AG. Balancing STING in antimicrobial defense and autoinflammation. Cytokine Growth Factor Rev (2020) 55:1–14. doi: 10.1016/j.cytogfr.2020.06.004 32563552

[26] Ablasser A Goldeck M Cavlar T Deimling T Witte G Röhl I. cGAS produces a 2’-5’-linked cyclic dinucleotide second messenger that activates STING. Nature (2013) 498:380–4. doi: 10.1038/nature12306 PMC4143541 23722158

[27] Zhang X Shi H Wu J Zhang X Sun L Chen C. Cyclic GMP-AMP containing mixed phosphodiester linkages is an endogenous high-affinity ligand for STING. Mol Cell (2013) 51:226–35. doi: 10.1016/j.molcel.2013.05.022 PMC3808999 23747010

[28] Zhou C Chen X Planells-Cases R Chu J Wang L Cao L. Transfer of cGAMP into Bystander Cells via LRRC8 Volume-Regulated Anion Channels Augments STING-Mediated Interferon Responses and Anti-viral Immunity. Immunity (2020) 52:767–81.e6. doi: 10.1016/j.immuni.2020.03.016 32277911

[29] Ablasser A Schmid-Burgk JL Hemmerling I Horvath GL Schmidt T Latz E. Cell intrinsic immunity spreads to bystander cells via the intercellular transfer of cGAMP. Nature (2013) 503:530–4. doi: 10.1038/nature12640 PMC4142317 24077100

[30] Dobbs N Burnaevskiy N Chen D Gonugunta VK Alto NM Yan N. STING activation by translocation from the ER is associated with infection and autoinflammatory disease. Cell Host Microbe (2015) 18:157–68. doi: 10.1016/j.chom.2015.07.001 PMC4537353 26235147

[31] Zhang X Wu J Du F Xu H Sun L Chen Z. The cytosolic DNA sensor cGAS forms an oligomeric complex with DNA and undergoes switch-like conformational changes in the activation loop. Cell Rep (2014) 6:421–30. doi: 10.1016/j.celrep.2014.01.003 PMC3969844 24462292

[32] Mukai K Konno H Akiba T Uemura T Waguri S Kobayashi T. Activation of STING requires palmitoylation at the Golgi. Nat Commun (2016) 7:11932. doi: 10.1038/ncomms11932 27324217 PMC4919521

[33] Mukai K Ogawa E Uematsu R Kuchitsu Y Kiku F Uemura T. Homeostatic regulation of STING by retrograde membrane traffic to the ER. Nat Commun (2021) 12:61. doi: 10.1038/s41467-020-20234-9 33397928 PMC7782846

[34] Steiner A Hrovat-Schaale K Prigione I Yu CH Laohamonthonkul P Harapas CR. Deficiency in coatomer complex I causes aberrant activation of STING signalling. Nat Commun (2022) 13:2321. doi: 10.1038/s41467-022-29946-6 35484149 PMC9051092

[35] Zhang C Shang G Gui X Zhang X Bai XC Chen ZJ. Structural basis of STING binding with and phosphorylation by TBK1. Nature (2019) 567:394–8. doi: 10.1038/s41586-019-1000-2 PMC6862768 30842653

[36] Liu S Cai X Wu J Cong Q Chen X Li T. Phosphorylation of innate immune adaptor proteins MAVS, STING, and TRIF induces IRF3 activation. Science (2015) 347:aaa2630. doi: 10.1126/science.aaa2630 25636800

[37] Zhao B Shu C Gao X Sankaran B Du F Shelton CL. Structural basis for concerted recruitment and activation of IRF-3 by innate immune adaptor proteins. Proc Natl Acad Sci USA (2016) 113:E3403–12. doi: 10.1073/pnas.1603269113 PMC4914169 27302953

[38] Ma X Helgason E Phung QT Quan CL Iyer RS Lee MW. Molecular basis of Tank-binding kinase 1 activation by transautophosphorylation. Proc Natl Acad Sci USA (2012) 109:9378–83. doi: 10.1073/pnas.1121552109 PMC3386122 22619329

[39] Agalioti T Lomvardas S Parekh B Yie J Maniatis T Thanos D. Ordered recruitment of chromatin modifying and general transcription factors to the IFN-beta promoter. Cell (2000) 103:667–78. doi: 10.1016/s0092-8674(00)00169-0 11106736

[40] Abe T Barber GN. Cytosolic-DNA-mediated, STING-dependent proinflammatory gene induction necessitates canonical NF-κB activation through TBK1. J Virol (2014) 88:5328–41. doi: 10.1128/JVI.00037-14 PMC4019140 24600004

[41] Bader CS Jin L Levy RB. STING and transplantation: can targeting this pathway improve outcomes? Blood (2021) 137:1871–8. doi: 10.1182/blood.2020008911 PMC8033456 33619537

[42] Chen X Wang Q Shao M Ma L Guo D Wu Y. Ginsenoside Rb3 regulates energy metabolism and apoptosis in cardiomyocytes via activating PPARα pathway. BioMed Pharmacother (2019) 120:109487. doi: 10.1016/j.biopha.2019.109487 31577975

[43] Sun J Yu X Huangpu H Yao F. Ginsenoside Rb3 protects cardiomyocytes against hypoxia/reoxygenation injury via activating the antioxidation signaling pathway of PERK/Nrf2/HMOX1. BioMed Pharmacother (2019) 109:254–61. doi: 10.1016/j.biopha.2018.09.002 30396083

[44] Li Y Liu H Zeng Z Lin H Chen X Yuan X. Ginsenoside Rb3 attenuates skin flap ischemia-reperfusion damage by inhibiting STING-IRF3 signaling. J Mol Histol (2022) 53:763–72. doi: 10.1007/s10735-022-10081-x 35732862

[45] Yang Y Ma Y Yu S Lin Z Yan C Wang Y. TIPE2 knockout reduces myocardial cell damage by inhibiting IFN-γ-mediated ferroptosis. Biochim Biophys Acta Mol Basis Dis (2023) 1869:166566. doi: 10.1016/j.bbadis.2022.166566 36216021

[46] Wang Q Wei C Ma L Wang X Li L Zhou Q. Inflammatory cytokine TNF-α promotes corneal endothelium apoptosis via upregulating TIPE2 transcription during corneal graft rejection. Graefes Arch Clin Exp Ophthalmol (2018) 256:709–15. doi: 10.1007/s00417-018-3913-0 29480366

[47] Almansoori AA Khentii N Kim B Kim SM Lee JH. Mesenchymal stem cell therapy in submandibular salivary gland allotransplantation: experimental study. Transplantation (2019) 103:1111–20. doi: 10.1097/TP.0000000000002612 30801515

[48] Dreyer GJ Groeneweg KE Heidt S Roelen DL van Pel M Roelofs H. Human leukocyte antigen selected allogeneic mesenchymal stromal cell therapy in renal transplantation: The Neptune study, a phase I single-center study. Am J Transplant (2020) 20:2905–15. doi: 10.1111/ajt.15910 PMC7586810 32277568

[49] Chen Y Yan G Ma Y Zhong M Yang Y Guo J. Combination of mesenchymal stem cells and FK506 prolongs heart allograft survival by inhibiting TBK1/IRF3-regulated-IFN-γ production. Immunol Lett (2021) 238:21–8. doi: 10.1016/j.imlet.2021.06.007 34228988

[50] Ishikawa T Tamura E Kasahara M Uchida H Higuchi M Kobayashi H. Severe liver disorder following liver transplantation in STING-associated vasculopathy with onset in infancy. J Clin Immunol (2021) 41:967–74. doi: 10.1007/s10875-021-00977-w 33544357

[51] Picard C Thouvenin G Kannengiesser C Dubus JC Jeremiah N Rieux-Laucat F. Severe pulmonary fibrosis as the first manifestation of interferonopathy (TMEM173 mutation). Chest (2016) 150:e65–71. doi: 10.1016/j.chest.2016.02.682 27613991

[52] Kim D Song KB Choi EJ Yu J. A 17-year-old girl diagnosed with STING-associated vasculopathy with onset in infancy (SAVI) after lung transplantation. Chest (2022) 162:e249–52. doi: 10.1016/j.chest.2022.05.025 36344133

[53] Li Q Lan P. Activation of immune signals during organ transplantation. Signal Transduct Target Ther (2023) 8:110. doi: 10.1038/s41392-023-01377-9 36906586 PMC10008588

[54] Capuzzimati M Hough O Liu M. Cell death and ischemia-reperfusion injury in lung transplantation. J Heart Lung Transplant (2022) 41:1003–13. doi: 10.1016/j.healun.2022.05.013 35710485

[55] Murthy AMV Robinson N Kumar S. Crosstalk between cGAS-STING signaling and cell death. Cell Death Differ (2020) 27:2989–3003. doi: 10.1038/s41418-020-00624-8 32948836 PMC7560597

[56] Li J Sun X Yang N Ni J Xie H Guo H. Phosphoglycerate mutase 5 initiates inflammation in acute kidney injury by triggering mitochondrial DNA release by dephosphorylating the pro-apoptotic protein Bax. Kidney Int (2023) 103:115–33. doi: 10.1016/j.kint.2022.08.022 36089186

[57] Feng Y Imam Aliagan A Tombo N Draeger D Bopassa JC. RIP3 Translocation into Mitochondria Promotes Mitofilin Degradation to Increase Inflammation and Kidney Injury after Renal Ischemia-Reperfusion. Cells (2022) 11:1894. doi: 10.3390/cells11121894 35741025 PMC9220894

[58] Xu J Wu D Zhou S Hu H Li F Guan Z. MLKL deficiency attenuated hepatocyte oxidative DNA damage by activating mitophagy to suppress macrophage cGAS-STING signaling during liver ischemia and reperfusion injury. Cell Death Discov (2023) 9:58. doi: 10.1038/s41420-023-01357-6 36765043 PMC9918524

[59] Kobritz M Borjas T Patel V Coppa G Aziz M Wang P. H151, A SMALL MOLECULE INHIBITOR OF STING AS A NOVEL THERAPEUTIC IN INTESTINAL ISCHEMIA-REPERFUSION INJURY. Shock (2022) 58:241–50. doi: 10.1097/SHK.0000000000001968 PMC9489661 35959789

[60] Liao Y Cheng J Kong X Li S Li X Zhang M. HDAC3 inhibition ameliorates ischemia/reperfusion-induced brain injury by regulating the microglial cGAS-STING pathway. Theranostics (2020) 10:9644–62. doi: 10.7150/thno.47651 PMC7449914 32863951

[61] Zhong W Rao Z Rao J Han G Wang P Jiang T. Aging aggravated liver ischemia and reperfusion injury by promoting STING-mediated NLRP3 activation in macrophages. Aging Cell (2020) 19:e13186. doi: 10.1111/acel.13186 32666684 PMC7431827

[62] Jiao J Jiang Y Qian Y Liu G Xu M Wang F. Expression of STING is increased in monocyte-derived macrophages and contributes to liver inflammation in hepatic ischemia-reperfusion injury. Am J Pathol (2022) 192:1745–62. doi: 10.1016/j.ajpath.2022.09.002 36174680

[63] Kong L Li W Chang E Wang W Shen N Xu X. mtDNA-STING axis mediates microglial polarization via IRF3/NF-κB signaling after ischemic stroke. Front Immunol (2022) 13:860977. doi: 10.3389/fimmu.2022.860977 35450066 PMC9017276

[64] Corrales L McWhirter SM Dubensky TW Jr Gajewski TF. The host STING pathway at the interface of cancer and immunity. J Clin Invest (2016) 126:2404–11. doi: 10.1172/JCI86892 PMC4922692 27367184

[65] Li S Xu H Song M Shaw BI Li QJ Kirk AD. IFI16-STING-NF-κB signaling controls exogenous mitochondrion-induced endothelial activation. Am J Transplant (2022) 22:1578–92. doi: 10.1111/ajt.17034 PMC9177674 35322536

[66] Huang R Shi Q Zhang S Lin H Han C Qian X. Inhibition of the cGAS-STING Pathway Attenuates Lung Ischemia/Reperfusion Injury via Regulating Endoplasmic Reticulum Stress in Alveolar Epithelial Type II Cells of Rats. J Inflamm Res (2022) 15:5103–19. doi: 10.2147/JIR.S365970 PMC9462969 36091334

[67] Zhan Y Xu D Tian Y Qu X Sheng M Lin Y. Novel role of macrophage TXNIP-mediated CYLD-NRF2-OASL1 axis in stress-induced liver inflammation and cell death. JHEP Rep (2022) 4:100532. doi: 10.1016/j.jhepr.2022.100532 36035360 PMC9404660

[68] Yang M Ma YX Zhi Y Wang HB Zhao L Wang PS. Inhibitors of IFN gene stimulators (STING) improve intestinal ischemia-reperfusion-induced acute lung injury by activating AMPK signaling. Eur J Med Res (2022) 27:79. doi: 10.1186/s40001-022-00703-1 35642042 PMC9153160

[69] Wu J Liu Q Zhang X Wu X Zhao Y Ren J. STING-dependent induction of lipid peroxidation mediates intestinal ischemia-reperfusion injury. Free Radic Biol Med (2021) 163:135–40. doi: 10.1016/j.freeradbiomed.2020.12.010 33347986

[70] Li JK Song ZP Hou XZ. Scutellarin ameliorates ischemia/reperfusion injury−induced cardiomyocyte apoptosis and cardiac dysfunction via inhibition of the cGAS−STING pathway. Exp Ther Med (2023) 25:155. doi: 10.3892/etm.2023.11854 36911381 PMC9996299

[71] Shen A Zheng D Luo Y Mou T Chen Q Huang Z. MicroRNA-24-3p alleviates hepatic ischemia and reperfusion injury in mice through the repression of STING signaling. Biochem Biophys Res Commun (2020) 522:47–52. doi: 10.1016/j.bbrc.2019.10.182 31735332

[72] Li B Wang W Li Y Wang S Liu H Xia Z. cGAS-STING pathway aggravates early cerebral ischemia-reperfusion injury in mice by activating NCOA4-mediated ferritinophagy. Exp Neurol (2023) 359:114269. doi: 10.1016/j.expneurol.2022.114269 36343680

[73] Liu M Li Y Han S Wang H Li J. Activin A alleviates neuronal injury through inhibiting cGAS-STING-mediated autophagy in mice with ischemic stroke. J Cereb Blood Flow Metab (2023) 43:736–48. doi: 10.1177/0271678X221147056 PMC10108189 36537048

[74] Lin F Yao X Kong C Liu X Zhao Z Rao S. 25-Hydroxycholesterol protecting from cerebral ischemia-reperfusion injury through the inhibition of STING activity. Aging (Albany NY) (2021) 13:20149–63. doi: 10.18632/aging.203337 PMC8436919 34406977

[75] Lei Z Deng M Yi Z Sun Q Shapiro RA Xu H. cGAS-mediated autophagy protects the liver from ischemia-reperfusion injury independently of STING. Am J Physiol Gastrointest Liver Physiol (2018) 314:G655–67. doi: 10.1152/ajpgi.00326.2017 PMC6032062 29446653

[76] Tang LJ Zhou YJ Xiong XM Li NS Zhang JJ Luo XJ. Ubiquitin-specific protease 7 promotes ferroptosis via activation of the p53/TfR1 pathway in the rat hearts after ischemia/reperfusion. Free Radic Biol Med (2021) 162:339–52. doi: 10.1016/j.freeradbiomed.2020.10.307 33157209

[77] Zhang X Wu J Liu Q Li X Li S Chen J. mtDNA-STING pathway promotes necroptosis-dependent enterocyte injury in intestinal ischemia reperfusion. Cell Death Dis (2020) 11:1050. doi: 10.1038/s41419-020-03239-6 33311495 PMC7732985

[78] Wu XY Chen YJ Liu CA Gong JH Xu XS. STING induces liver ischemia-reperfusion injury by promoting calcium-dependent caspase 1-GSDMD processing in macrophages. Oxid Med Cell Longev (2022) 2022:8123157. doi: 10.1155/2022/8123157 35281468 PMC8906939

[79] Mallavia B Liu F Lefrançais E Cleary SJ Kwaan N Tian JJ. Mitochondrial DNA stimulates TLR9-dependent neutrophil extracellular trap formation in primary graft dysfunction. Am J Respir Cell Mol Biol (2020) 62:364–72. doi: 10.1165/rcmb.2019-0140OC PMC7055700 31647878

[80] Xian H Watari K Sanchez-Lopez E Offenberger J Onyuru J Sampath H. Oxidized DNA fragments exit mitochondria via mPTP- and VDAC-dependent channels to activate NLRP3 inflammasome and interferon signaling. Immunity (2022) 55:1370–85.e8. doi: 10.1016/j.immuni.2022.06.007 35835107 PMC9378606

[81] McArthur K Whitehead LW Heddleston JM Li L Padman BS Oorschot V. BAK/BAX macropores facilitate mitochondrial herniation and mtDNA efflux during apoptosis. Science (2018) 359:eaao6047. doi: 10.1126/science.aao6047 29472455

[82] Riley JS Quarato G Cloix C Lopez J O’Prey J Pearson M. Mitochondrial inner membrane permeabilisation enables mtDNA release during apoptosis. EMBO J (2018) 37:e99238. doi: 10.15252/embj.201899238 30049712 PMC6120664

[83] Konno H Konno K Barber GN. Cyclic dinucleotides trigger ULK1 (ATG1) phosphorylation of STING to prevent sustained innate immune signaling. Cell (2013) 155:688–98. doi: 10.1016/j.cell.2013.09.049 PMC3881181 24119841

[84] Xia P Ye B Wang S Zhu X Du Y Xiong Z. Glutamylation of the DNA sensor cGAS regulates its binding and synthase activity in antiviral immunity. Nat Immunol (2016) 17:369–78. doi: 10.1038/ni.3356 26829768

[85] Panzer SE. Macrophages in transplantation: A matter of plasticity, polarization, and diversity. Transplantation (2022) 106:257–67. doi: 10.1097/TP.0000000000003804 PMC8548398 33908380

[86] Lan YY Heather JM Eisenhaure T Garris CS Lieb D Raychowdhury R. Extranuclear DNA accumulates in aged cells and contributes to senescence and inflammation. Aging Cell (2019) 18:e12901. doi: 10.1111/acel.12901 30706626 PMC6413746

[87] Kanneganti M Olthoff KM Bittermann T. Impact of older donor age on recipient and graft survival after LDLT: the US experience. Transplantation (2023) 107:162–71. doi: 10.1097/TP.0000000000004289 PMC9771867 36042545

[88] Wu Z Miao X Jiang Y Kong D Liu H Xie W. Cardiomyocytic cyclic GMP-AMP synthase is critical for the induction of experimental cardiac graft rejection. J Thorac Cardiovasc Surg (2023), S0022–522300199-X. doi: 10.1016/j.jtcvs.2023.03.005 37061907

[89] Field CS Baixauli F Kyle RL Puleston DJ Cameron AM Sanin DE. Mitochondrial integrity regulated by lipid metabolism is a cell-intrinsic checkpoint for treg suppressive function. Cell Metab (2020) 31:422–437.e5. doi: 10.1016/j.cmet.2019.11.021 31883840 PMC7001036

[90] Luksch H Stinson WA Platt DJ Qian W Kalugotla G Miner CA. STING-associated lung disease in mice relies on T cells but not type I interferon. J Allergy Clin Immunol (2019) 144:254–266.e8. doi: 10.1016/j.jaci.2019.01.044 30772497 PMC6612314

[91] Kariya T Ueta H Xu XD Koga D Ezaki T Yu E. Direct evidence for activated CD8+ T cell transmigration across portal vein endothelial cells in liver graft rejection. J Gastroenterol (2016) 51:985–98. doi: 10.1007/s00535-016-1169-1 PMC5037149 26891909

[92] Lin L Xu H Bishawi M Feng F Samy K Truskey G. Circulating mitochondria in organ donors promote allograft rejection. Am J Transplant (2019) 19:1917–29. doi: 10.1111/ajt.15309 PMC6591073 30761731

[93] Cloer CM Givens CS Buie LK Rochelle LK Lin YT Popa S. Mitochondrial transplant after ischemia reperfusion promotes cellular salvage and improves lung function during ex-vivo lung perfusion. J Heart Lung Transplant (2023) 42:575–84. doi: 10.1016/j.healun.2023.01.002 36707296

[94] Namgaladze D Khodzhaeva V Brüne B. ER-mitochondria communication in cells of the innate immune system. Cells (2019) 8:1088. doi: 10.3390/cells8091088 31540165 PMC6770024

[95] Pereira AC De Pascale J Resende R Cardoso S Ferreira I Neves BM. ER-mitochondria communication is involved in NLRP3 inflammasome activation under stress conditions in the innate immune system. Cell Mol Life Sci (2022) 79:213. doi: 10.1007/s00018-022-04211-7 35344105 PMC11072401

[96] Hasan M Gonugunta VK Dobbs N Ali A Palchik G Calvaruso MA. Chronic innate immune activation of TBK1 suppresses mTORC1 activity and dysregulates cellular metabolism. Proc Natl Acad Sci USA (2017) 114:746–51. doi: 10.1073/pnas.1611113114 PMC5278463 28069950

[97] Zhao P Wong KI Sun X Reilly SM Uhm M Liao Z. TBK1 at the crossroads of inflammation and energy homeostasis in adipose tissue. Cell (2018) 172:731–43.e12. doi: 10.1016/j.cell.2018.01.007 29425491 PMC5808582

[98] Sanz MN Farine E Niederberger P Méndez-Carmona N Wyss RK Arnold M. Cardioprotective reperfusion strategies differentially affect mitochondria: Studies in an isolated rat heart model of donation after circulatory death (DCD). Am J Transplant (2019) 19:331–44. doi: 10.1111/ajt.15024 30019521

[99] Tang CH Zundell JA Ranatunga S Lin C Nefedova Y Del Valle JR. Agonist-mediated activation of STING induces apoptosis in Malignant B cells. Cancer Res (2016) 76:2137–52. doi: 10.1158/0008-5472.CAN-15-1885 PMC4873432 26951929

[100] Gulen MF Koch U Haag SM Schuler F Apetoh L Villunger A. Signalling strength determines proapoptotic functions of STING. Nat Commun (2017) 8:427. doi: 10.1038/s41467-017-00573-w 28874664 PMC5585373

[101] Xu LJ Chen RC Ma XY Zhu Y Sun GB Sun XB. Scutellarin protects against myocardial ischemia-reperfusion injury by suppressing NLRP3 inflammasome activation. Phytomedicine (2020) 68:153169. doi: 10.1016/j.phymed.2020.153169 31999976

[102] Xiao X Lu Z Lin V May A Shaw DH Wang Z. MicroRNA miR-24-3p reduces apoptosis and regulates keap1-nrf2 pathway in mouse cardiomyocytes responding to ischemia/reperfusion injury. Oxid Med Cell Longev (2018) 2018:7042105. doi: 10.1155/2018/7042105 30622671 PMC6304907

[103] Bardhi E McDaniels J Rousselle T Maluf DG Mas VR. Nucleic acid biomarkers to assess graft injury after liver transplantation. JHEP Rep (2022) 4:100439. doi: 10.1016/j.jhepr.2022.100439 35243279 PMC8856989

[104] Mao B Yuan W Wu F Yan Y Wang B. Autophagy in hepatic ischemia-reperfusion injury. Cell Death Discov (2023) 9:115. doi: 10.1038/s41420-023-01387-0 37019879 PMC10076300

[105] Cao Q Liu Z Xiong Y Zhong Z Ye Q. Multiple roles of 25-hydroxycholesterol in lipid metabolism, antivirus process, inflammatory response, and cell survival. Oxid Med Cell Longev (2020) 2020:8893305. doi: 10.1155/2020/8893305 33274010 PMC7695496

[106] Chen C Xu P. Cellular functions of cGAS-STING signaling. Trends Cell Biol (2022) 33:630–48. doi: 10.1016/j.tcb.2022.11.001 36437149

[107] Li C Zhang Y Liu J Kang R Klionsky DJ Tang D. Mitochondrial DNA stress triggers autophagy-dependent ferroptotic death. Autophagy (2021) 17:948–60. doi: 10.1080/15548627.2020.1739447 PMC8078708 32186434

[108] Yu Y Yan Y Niu F Wang Y Chen X Su G. Ferroptosis: a cell death connecting oxidative stress, inflammation and cardiovascular diseases. Cell Death Discov (2021) 7:193. doi: 10.1038/s41420-021-00579-w 34312370 PMC8313570

[109] Li W Feng G Gauthier JM Lokshina I Higashikubo R Evans S. Ferroptotic cell death and TLR4/Trif signaling initiate neutrophil recruitment after heart transplantation. J Clin Invest (2019) 129:2293–304. doi: 10.1172/JCI126428 PMC6546457 30830879

[110] Jia M Qin D Zhao C Chai L Yu Z Wang W. Redox homeostasis maintained by GPX4 facilitates STING activation. Nat Immunol (2020) 21:727–35. doi: 10.1038/s41590-020-0699-0 32541831

[111] Dong B Ding C Xiang H Zheng J Li X Xue W. USP7 accelerates FMR1-mediated ferroptosis by facilitating TBK1 ubiquitination and DNMT1 deubiquitination after renal ischemia-reperfusion injury. Inflamm Res (2022) 71:1519–33. doi: 10.1007/s00011-022-01648-1 36264362

[112] Lukenaite B Griciune E Leber B Strupas K Stiegler P Schemmer P. Necroptosis in solid organ transplantation: A literature overview. Int J Mol Sci (2022) 23:3677. doi: 10.3390/ijms23073677 35409037 PMC8998671

[113] Lucas-Ruiz F Peñín-Franch A Pons JA Ramírez P Pelegrín P Cuevas S. Emerging role of NLRP3 inflammasome and pyroptosis in liver transplantation. Int J Mol Sci (2022) 23:14396. doi: 10.3390/ijms232214396 36430874 PMC9698208

[114] Sarhan J Liu BC Muendlein HI Weindel CG Smirnova I Tang AY. Constitutive interferon signaling maintains critical threshold of MLKL expression to license necroptosis. Cell Death Differ (2019) 26:332–47. doi: 10.1038/s41418-018-0122-7 PMC6329789 29786074

[115] Gaidt MM Ebert TS Chauhan D Ramshorn K Pinci F Zuber S. The DNA inflammasome in human myeloid cells is initiated by a STING-cell death program upstream of NLRP3. Cell (2017) 171:1110–24.e18. doi: 10.1016/j.cell.2017.09.039 29033128 PMC5901709

[116] Roberts MB Fishman JA. Immunosuppressive agents and infectious risk in transplantation: managing the “Net state of immunosuppression”. Clin Infect Dis (2021) 73:e1302–17. doi: 10.1093/cid/ciaa1189 PMC8561260 32803228

[117] Jansen MPB Pulskens WPC Uil M Claessen N Nieuwenhuizen G Standaar D. Urinary mitochondrial DNA associates with delayed graft function following renal transplantation. Nephrol Dial Transplant (2020) 35:1320–7. doi: 10.1093/ndt/gfy372 30590723

[118] Miner JJ Fitzgerald KA. A path towards personalized medicine for autoinflammatory and related diseases. Nat Rev Rheumatol (2023) 19:182–9. doi: 10.1038/s41584-022-00904-2 PMC9904876 36750685

